# Differentiated HT22 cells as a novel model for *in vitro* screening of serotonin reuptake inhibitors

**DOI:** 10.3389/fphar.2022.1062650

**Published:** 2023-01-10

**Authors:** Juhee Lim, Yeojin Bang, Kyeong-Man Kim, Hyun Jin Choi

**Affiliations:** ^1^ College of Pharmacy and Institute of Pharmaceutical Sciences, CHA University, Pocheon, Gyeonggi-do, South Korea; ^2^ College of Pharmacy and Research Institute of Pharmaceutical Sciences, Woosuk University, Wanju, Jeollabuk-do, South Korea; ^3^ College of Pharmacy, Chonnam National University, Gwangju, South Korea

**Keywords:** HT22, differentiation, serotonin transporter, serotonin reuptake inhibitor, Pet-1

## Abstract

The mouse hippocampal neuronal cell line HT22 is frequently used as an *in vitro* model to investigate the role of hippocampal cholinergic neurons in cognitive functions. HT22 cells are derived from hippocampal neuronal HT4 cells. However, whether these cells exhibit the serotonergic neuronal phenotype observed in mature hippocampal neurons has not been determined yet. In this present study, we examined whether the differentiation of HT22 cells enhances the serotonergic neuronal phenotype, and if so, whether it can be used for antidepressant screening. Our results show that differentiation of HT22 cells promoted neurite outgrowth and upregulation of N-methyl-D-aspartate receptor and choline acetyltransferase, which is similar to that observed in primary cultured hippocampal neurons. Furthermore, proteins required for serotonergic neurotransmission, such as tryptophan hydroxylase 2, serotonin (5-hydroxytryptamine, 5-HT)_1a_ receptor, and serotonin transporter (SERT), were significantly upregulated in differentiated HT22 cells. The transcription factor Pet-1 was upregulated during HT22 differentiation and was responsible for the regulation of the serotonergic neuronal phenotype. Differentiation also enhanced the functional serotonergic properties of HT22 cells, as evidenced by increase in intracellular 5-HT levels, serotonin transporter SERT glycosylation, and 5-HT reuptake activity. The sensitivity of 5-HT reuptake inhibition by venlafaxine in differentiated HT22 cells (IC_50,_ 27.21 nM) was comparable to that in HEK293 cells overexpressing serotonin transporter SERT (IC_50,_ 30.65 nM). These findings suggest that the differentiation of HT22 cells enhances their functional serotonergic properties, and these cells could be a potential *in vitro* system for assessing the efficacy of antidepressant 5-HT reuptake inhibitors.

## 1 Introduction

The monoamine hypothesis of depression states that a lack or imbalance of serotonin (5-hydroxytryptamine, 5-HT), norepinephrine, and/or dopamine in the central nervous system (CNS) is the pathophysiological basis for depression (Boku et al., 2018). Although the monoamine theory does not provide explain the extent of antidepressant effect, including delayed onset of response after antidepressant administration or recurrence of depressive symptoms despite antidepressant therapy, it is by far the most comprehendible hypothesis (Perez-Caballero et al., 2019). Based on the monoamine theory, the primary goal of antidepressants is to elevate monoamine levels in the synapse mainly by inhibiting monoamine reuptake. Selective serotonin reuptake inhibitors (SSRIs) and serotonin norepinephrine reuptake inhibitors (SNRIs) are two major clinical antidepressants used to relieve the symptoms of depression and anxiety (Baudry et al., 2019). Both drugs target and inhibit the serotonin transporter (SERT), which is an integral membrane transporter that reuptakes 5-HT from the synaptic cleft and transports it back to the presynaptic neuron. Therefore, the inhibitory effect of drug candidates on 5-HT uptake *via* SERT is an important indicator of their antidepressant efficacy. SERT-overexpressing HEK293 (HEK293-SERT) cells have been commonly used as an *in vitro* system to measure 5-HT uptake activity through SERT (Manepalli et al., 2011; Yahata et al., 2017). HEK293-SERT cells have the advantage of expressing a pure population of a single human SERT, but could be subjective to specific experimental method-dependent variability, which can lead to high variability in transporter inhibition potency values (Ilic et al., 2020). In addition, SERT function can be regulated by several cellular factors in neurons, that is, different regulations of SERT by vesicle-associated membrane protein 2 between neuronal and non-neuronal origin. Therefore, HEK293-SERT cells may not accurately reflect the physiological state of neurons in the complex microenvironment of the CNS (Muller et al., 2014).

The hippocampus is a complex region in the temporal lobes of the brain that is associated with emotional and cognitive functions (Battaglia et al., 2011). Brain imaging studies have reported that reduced hippocampal volume is a critical feature of major depressive disorders (Malykhin et al., 2010; Opel et al., 2014). In particular, in women with depression, the severity and duration of depressive symptoms are linked to a reduction in hippocampal volume (Elbejjani et al., 2015). Although the reason for this reduction in hippocampal volume in patients with depression is unclear, recent studies suggest the neuroplasticity hypothesis, which focuses on the morphological changes of neurons, and the neurogenesis hypothesis, focuses on decreased hippocampal neurogenesis (Lim et al., 2018; Bang et al., 2021). The hippocampus is densely innervated by serotonergic fibers, therefore, it contains characteristic proteins involved in serotonergic neurotransmission, such as 5-HT synthesizing enzymes and specific receptors, and SERT. Almost all 5-HT receptor subtypes are expressed in the hippocampus as presynaptic and postsynaptic receptors, and hippocampal neurons receive extensive serotonergic input, notably from the raphe nucleus (Berumen et al., 2012). Therefore, the identification of the pathophysiology of depression and the mechanism of action of antidepressants requires a comprehensive study of various characteristics of the hippocampal neurons, including neurite outgrowth, synapse formation, neuronal development, and 5-HT transmission.

HT22 cells, which are derived from parent HT4 cells that are immortalized from primary mouse hippocampal neuronal cells (Morimoto and Koshland, 1990), are generally used in research related to cognitive function and associated disorders, such as Alzheimer’s disease (Chen et al., 2020; Ma et al., 2020). Unlike mature hippocampal neurons, undifferentiated HT22 cells express very low levels of cholinergic markers and glutamate receptors (Liu et al., 2009; He et al., 2013). However, differentiation of HT22 cells results in increased levels of N-methyl-d-aspartate receptor (NMDAR) mRNA (Zhao et al., 2012), making them more susceptible to glutamate-induced excitotoxicity (He et al., 2013). Moreover, differentiated HT22 cells express higher levels of molecules essential for cholinergic neurons, such as choline acetyltransferase (ChAT), choline transporters, and muscarinic acetylcholine receptors, and appear to have cholinergic neuron-like morphology and biochemistry (Liu et al., 2009). Because HT22 cells are derived from hippocampal neuronal cell lines, they may exhibit the serotonergic neuronal phenotype seen in mature hippocampal neurons; however, this has not been reported.

Therefore, in this study, we examined the serotonergic properties of differentiated HT22 cells. We show that the differentiation of HT22 cells enhances the expression of NMDAR and cholinergic markers, as well as molecules essential for serotonergic neurons, SERT, tryptophan hydroxylase 2 (TPH2), and 5-HT_1a_R. Furthermore, 5-HT uptake *via* SERT in differentiated HT22 cells was as high as that in HEK293-SERT cells. These results suggest that differentiated HT22 cells could be a novel *in vitro* model for the development of drugs that target serotonergic neurotransmission.

## 2 Methods

### 2.1 Antibodies and reagents

Neurobasal medium (21103-049), L-glutamine (25030-081), N-2 supplement (100X) (17502-048), B27 supplement (50X) (17504-044), laminin (23017-015) and cell dissociation reagent (A1110501) were obtained from Gibco (Grand Island, NY, United States). Dulbecco’s modified Eagle’s medium (DMEM) (10-013-CV), fetal bovine serum (FBS) (35-015-CV) and penicillin/streptomycin (30-002-CI) were purchased from Corning (Corning, NY, United States), and 4′6-diamidino-2-phenylindole dilactate (DAPI) (D3571) was obtained from Invitrogen (Waltham, MA, United States). Poly-d-lysine (P6407), 4-(4-(dimethylamino)phenyl)-1-methylpyridinium iodide (APP) (SML0756) were purchased from Sigma-Aldrich (St. Louis, MO, United States). Serotonin ELISA Kit (ab133053) was purchased from Abcam (Cambridge, UK). The following antibodies were used in this study: anti-5-HT_1a_R (GTX104703), anti-brain-derived neurotrophic factor (BDNF) (GTX132621), anti-SERT (GTX133101) (all from GeneTex, Irvine, CA, United States); anti-TPH2 (NB100-74555, Novus Biologicals; Littleton, CO, United States); anti-NMDA1R (#32-0500, Thermo Fisher Scientific, Pittsburgh, PA, United States); anti-Pet-1 (MyBioSource, San Diego, CA, United States); anti-ChAT (SC-55557) and anti-vinculin (SC-73614) (both from Santa Cruz Biotechnology, Dallas, TX, United States); anti-glycosylated SERT (AB9726, Millipore, Burlington, MA, United States); anti-GAPDH (#2118, Cell Signaling Technology, Danvers, MA, United States); anti-β-actin (A1978, Sigma-Aldrich); horseradish peroxidase (HRP)-conjugated goat anti-mouse IgG (H + L) (#31430) and goat anti-rabbit IgG (H + L) (#31460) secondary antibodies (both from Thermo Fisher Scientific.); Alexa Fluor^®^ conjugated goat-anti-mouse (A-11001 and A-11005) and goat-anti-rabbit IgG (A-11008 and A-11012) (all from Invitrogen).

### 2.2 Cell culture

HT22 cells and human embryonic kidney 293 (HEK293) cells were obtained from ATCC (Rockville, MD, United States) and grown in DMEM (Corning) supplemented with 10% FBS (Corning), 100 IU/L penicillin (Corning), and 10 μg/mL streptomycin (Corning) at 37°C in 5% CO_2_. The cells were placed on culture plates or dishes (Thermo Fisher Scientific) and were fed fresh medium after 24°h, for subsequent experiments. HT22 differentiation was performed in accordance with previous literature (He et al., 2013), with a slight modification (Neurobasal medium containing 2°mmol/L-glutamine and .5× N2 supplement) (Gibco).

Primary isolated hippocampal neurons from mice were obtained E18 embryo brains. One C57BL/6 pregnant female mouse was sacrificed for each culture and 8–10 E18 embryos were used (Orient Bio, Korea). For primary cultures of hippocampal neurons, removed the meninges from the medial aspect of the cerebral hemispheres with a dissecting microscope, then dissect out the hippocampus (Kaech and Banker, 2006). After dissection, the tissues were incubated at 37°C for 15 min in a cell dissociation reagent (Gibco) and washed with warm Hank’s balanced salt solution (Gibco). The tissues were mechanically triturated and resuspended in Neurobasal medium (Gibco) containing B27 supplement (50×; Gibco), L-glutamine (100×; Gibco), and penicillin/streptomycin solution (100×; Corning). The cells were maintained at 37°C and 5% CO_2_. All animal experiments were performed in accordance with the guidelines of the Institutional Animal Care and Use Committee (IACUC) of the CHA University (IACUC 200089).

### 2.3 Western blotting analysis

Assays were performed as described previously (Moon et al., 2022), with slight modifications. HT22 cells were washed with PBS and incubated with lysis buffer (RIPA buffer containing protease inhibitor and phosphatase inhibitor cocktails (Roche, Basel, Switzerland) for 30 min on ice. The cellular lysates were centrifuged at 21,206 g for 30 min at 4°C. The supernatant proteins were collected and total amount of protein in each samples was quantified using Bradford protein assay dye reagent (#5000006, Bio-Rad, Hercules, CA, United States). Calculated the average absorbance for each bovine serum albumin (BSA) standard and samples using the absorbance values at 595 nm using a microplatereader (VersaMax; Molecular Devices, San Jose, CA, United States). Equal amounts of protein (10–30 μg) were separated using sodium dodecyl sulfate polyacrylamide gel electrophoresis and transferred to polyvinylidene difluoride membranes (IPVH00010, Millipore, Burlington, MA, United States). The membranes were incubated with 5% skim milk (#232100, Thermo Fisher Scientific) diluting by 1X Tris buffered saline/Tween-20 (TBST) for 1 h. Then the specific primary antibodies including NMDA1R, ChAT, BDNF, SERT, 5-HT1aR and TPH2 (dilution 1:1,000), Pet-1 (dilution 1:2000), GAPDH (dilution 1:5,000), β-actin and vinculin (dilution 1:10000) were incubated at 4°C overnight. The HRP-conjugated goat anti-mouse IgG (H + L) and goat anti-rabbit IgG (H + L) secondary antibodies (dilution 1:2000 and 1:10000, respectively) were incubated at room temperature for 1 h. Specific proteins were visualized using enhanced chemiluminescent detection kits (WBKLS0500, Millipore) and analyzed using a luminescent image analyzer, ImageQuant LAS-4000 (Fujifilm, Tokyo, Japan), and Molecular Imager Gel Doc XR + System (Bio-Rad). GAPDH, vinculin, and β-actin were used as loading controls and the membrane was cut into several pieces to detect the target protein and the loading control protein simultaneously. Densitometric analysis was performed for each band obtained from all replicated experiment using ImageJ (National Institutes of Health, Bethesda, MD, United States), Multigauge (Fujifilm), and Image Lab software (Bio-Rad). Data are representative of at least three independent experiments and are quantified using densitometric analysis.

### 2.4 Small interfering RNA (siRNA) transfection

HT22 cells were placed in 6-well culture plates. The cells were transiently transfected with control siRNA (AM4635, Thermo Fisher Scientific) and two different types of Pet-1 siRNA (AM16706 and AM 16708, Thermo Fisher Scientific) after 24 h according to the manufacturer’s protocol with slight modification (Superfect^®^, Qiagen, Hilden, Germany). After 1 h of siRNA transfection, the cells were incubated for 2 h with normal growth medium, which was then replaced with fresh normal growth medium and differentiated medium for 3 days. Western blot analysis was performed on lysed HT22 proteins.

### 2.5 Immunofluorescence confocal microscopy

The primary cultures of hippocampal neurons and HT22 cells were plated on poly d-lysine (Sigma-Aldrich) and laminin (Gibco)-coated coverslips in 12-well culture plates. The cells were fixed for 15 min in cold 4% paraformaldehyde and 4% sucrose in PBS (pH 7.4). After washing in PBS containing 1% BSA, the cells were permeabilized for 10 min with .25% Triton X-100, then blocked with 1% BSA, 1% normal goat serum, and 3% FBS in PBS for 30 min at room temperature. The appropriate primary antibodies were applied to the cells and incubated at 4°C overnight. The primary antibodies used in this study are as follows: anti-NMDA1R (1:200), anti-ChAT (1:400), anti-BDNF (1:400), anti-SERT (1:100), anti-5-HT1aR (1:400), anti-TPH2 (1:400), and anti-pet-1 (1:400). After washing twice with 1% BSA in PBS, the cells were incubated with Alexa Fluor^®^ conjugated secondary antibodies (Invitrogen) for 1 h at room temperature, and DAPI (Invitrogen) was added for the final 10 min. The samples were mounted using Fluoromount/Plus mounting media (Diagnostic Biosystems, Pleasanton, CA, United States of America) for imaging, and images were obtained using a Zeiss LSM 880 confocal microscope (Carl Zeiss, Oberkochen, Germany).

### 2.6 Enzyme-linked immunosorbent assay (ELISA)

HT22 cells were placed on culture plates. After 24 h, they were fed fresh normal growth medium and differentiation medium for 3 days. The 5-HT levels in the cell lysates and culture medium were measured using a commercial ELISA kit (ab133053, Abcam), according to the manufacturer’s protocol. Assay buffer was used to dilute cellular supernatant and meium samples in a 1:16 ratio. The labeled alkaline-phosphatase conjugate and serotonin antibody were added to the samples and standards in duplicate. The absorbance at 405 nm of the resulting yellow solution was measured after the addition of a para-nitrophenyl phosphate substrate. According to a standard curve, absorbance (405 nm) was inversely proportional to serotonin concentrations. The results are presented as averages.

### 2.7 4-(4-(Dimethylamino)phenyl)-1-methylpyridinium iodide (APP)^+^ uptake assay

SERT activity in live HT22 cells was examined using APP^**+**^, which is a fluorescent neurotransmitter substrate (SML0756, Sigma-Aldrich). APP^**+**^ is nonfluorescent in solution, but fluoresces as the substrate accumulates, facilitating real-time kinetic evaluation of SERT activity. HT22 cells were placed in black 96-well microplates (655097, Greiner Bio-One GmbH, Kremsmuenster, Austria). After 24 h, they were fed fresh normal growth medium and differentiated medium. After 3 days, the cells were washed with PBS, and Krebs–Ringer solution (K4002, Sigma-Aldrich) containing 10 μM APP^**+**^ was added, and accumulated APP^**+**^ fluorescence was measured at 37°C for 40 min using a fluorescence microplate reader (Synergy Mx, BioTek, Winooski, VT, United States) at λex = 475 nm and λem = 605 nm. Five-HT competition experiments were performed by replacing the Krebs–Ringer solution containing fluoxetine (10 μM, 14418, Cayman chemical, Ann Arbor, MI, United States) and APP^**+**^ (10 μM).

### 2.8 5-HT reuptake assay

Assays were performed as described previously (Paudel et al., 2015), with slight modifications. HEK293 cells were cultured in a medium supplemented with FBS and transfected with human SERT. HT22 cells were cultured in a Neurobasal medium containing 2 mmol/L glutamine and .5× N2 supplement for 3 days. The medium was removed and cells were added uptake buffer (5 mM Tris base, 7.5 mM HEPES, 120 mM NaCl, 5.4 mM KCl, 1.2 mM CaCl2, 1.2 mM MgSO4, 1 mM ascorbic acid and 5 mM glucose; pH 7.1). After adding venlafaxine (.1 nM to 1 μM) to the wells, the 24-well plate was incubated at 37°C in a slide warmer (Fisher, Pittsburgh, PA, United States). Radiolabeled [^3^H]-5-HT (PerkinElmer, Waltham, MA, United States) was added (final concentration/well, 20 nM) and incubated for about 5 min at 37°C using a slide warmer (Fisher). The prepared cells were then lysed with 1% sodium dodecyl sulfate for about 2 h after being washed three times with ice-cold uptake buffer. The radioactivity was measured using a Wallac 1,450 MicroBeta^®^ TriLux liquid scintillation counter (PerkinElmer). Venlafaxine was used as the reference inhibitor of 5-HT reuptake.

### 2.9 Data analysis

All data were analyzed and expressed as the mean ± standard error of the mean (SEM). Comparisons between groups were obtained using one-way analysis of variance (ANOVA) with Tukey *post hoc* test or unpaired t-test of the Prism software (GraphPad, San Diego, CA, United States). *p*-values of *p < .05, **p < .01, ***p < .001, and ****p < .0001 were considered statistically significant.

## 3 Results

### 3.1 Differentiated HT22 cells have characteristics similar to those of hippocampal primary cells

**FIGURE 1:**
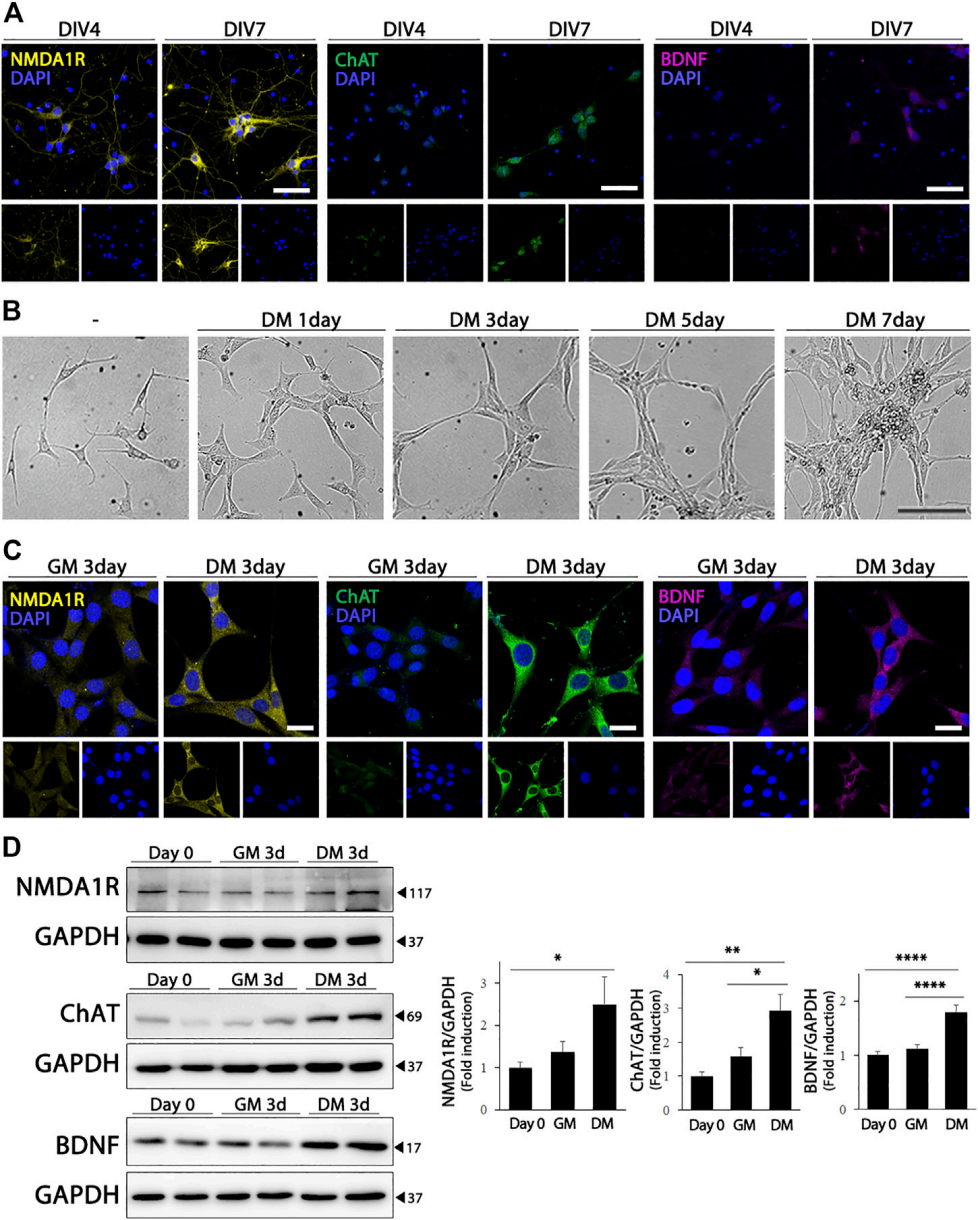
Cholinergic characteristics of differentiated HT22 cells. **(A)** Immunofluorescence staining of NMDA1R (yellow), ChAT (green), and BDNF (magenta) in hippocampal primary culture. DAPI staining (blue) indicates the nucleus. Scale bar = 50 μm. **(B)** Phase contrast image of differentiated HT22 cells according to time. Scale bar = 100 μm. **(C,D)** NMDA1R, ChAT, and BDNF immunofluorescence staining **(C)** and western blotting **(D)** of HT22 cells grown in normal growth medium and differentiation medium for 3 d. DAPI staining (blue) indicates the nucleus. Scale bar = 25 μm, GAPDH was used as loading control. Data are representative of at least three independent experiments and are quantified using densitometric analysis. Statistical significance: *p < .05, **p < .01, and ****p < .0001.

Primary cultures of mouse hippocampal neurons were used to study the physiological and pathological properties of the hippocampus. As shown in Figure 1A, the differentiation of hippocampal primary cultured neurons resulted in a significant increase in the expression of NMDA1R and ChAT. Brain-derived neurotrophic factor (BDNF), which is required for the development and differentiation of neurons, was also upregulated in primary hippocampal neurons. HT22 cells, which is an immortalized cell line derived from primary mouse hippocampal neurons, express very low levels of glutamate receptors and cholinergic markers in their undifferentiated states (Liu et al., 2009; He et al., 2013). In the differentiation medium, HT22 cells showed morphological changes similar to those of mature neurons, such as an increase in the number and length of cellular neurites (Figure 1B). Furthermore, differentiated HT22 cells expressed significantly higher levels of NMDA1R (F (2, 21) = 3.473, *p* = .0498), ChAT (F (2, 21) = 9.311, *p* = .0013) and BDNF (F (2, 33) = 21.96, *p* < .0001) than cells grown in normal growth medium (Figures 1C,D). These findings demonstrate that differentiated HT22 cells exhibit phenotypes similar to those of hippocampal primary neurons.

### 3.2 Differentiation of HT22 cells enhances serotonergic markers through Pet-1

**FIGURE 2:**
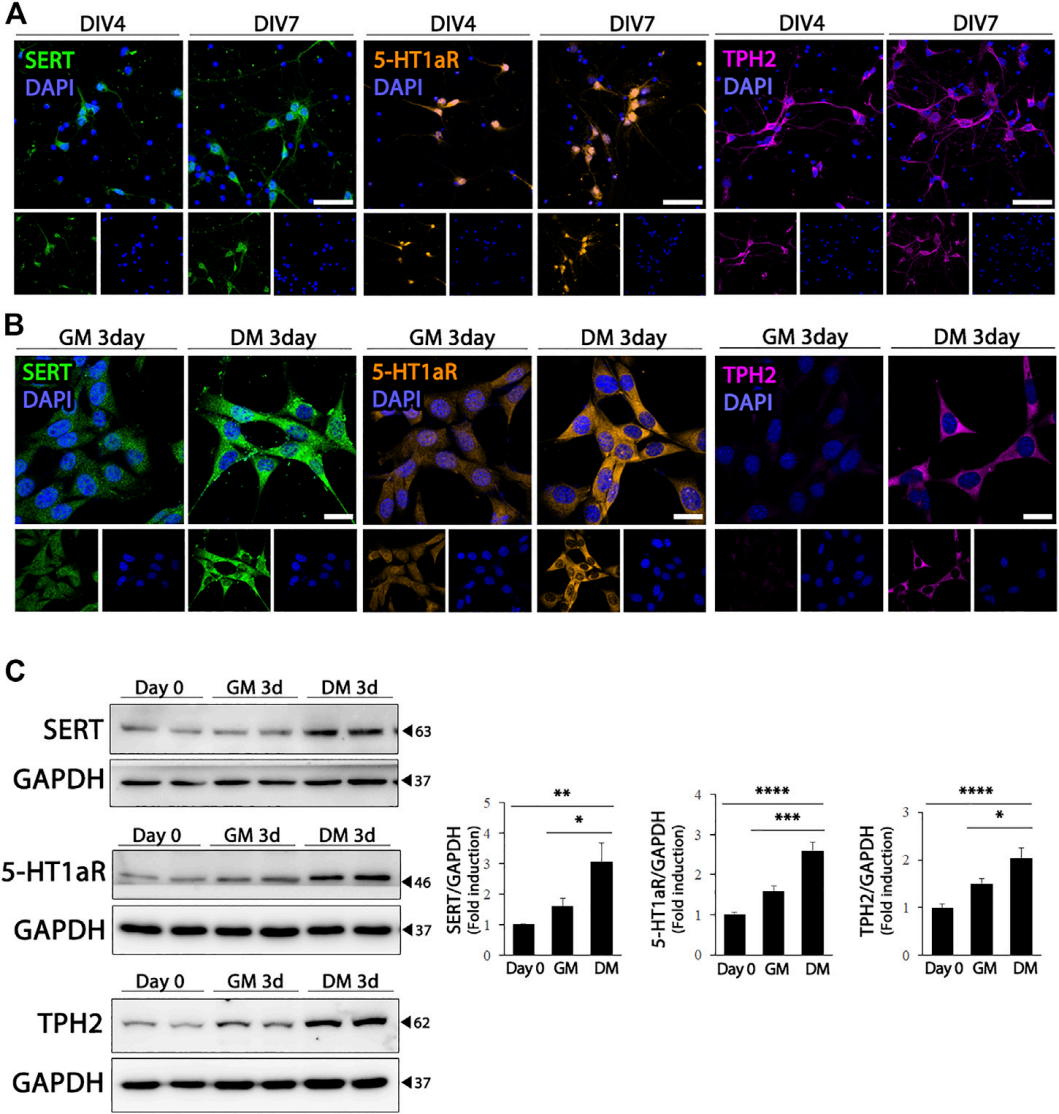
Enhanced serotonergic phenotypes of differentiated HT22 cells. **(A)** Immunofluorescence staining of SERT (green), 5-HT_1a_R (orange) and TPH2 (magenta) in hippocampal primary culture. DAPI staining (blue) indicates the nucleus. Scale bar = 50 μm. **(B,C)** SERT (green), 5-HT_1a_R (orange) and TPH2 (magenta) immunofluorescence staining **(B)** and western blotting **(C)** of HT22 cells grown in normal growth medium and differentiation medium for 3 d. DAPI staining (blue) indicates the nucleus. Scale bar = 25 μm. GAPDH was used as loading control. Data are representative of at least three independent experiments and are quantified using densitometric analysis. Statistical significance: *p < 0.05, **p < 0.01, ***p < 0.001 and ****p < 0.0001.

The hippocampus receives extensive serotonergic innervation. Therefore, we evaluated the effect of differentiation on the expression of serotonergic neuronal markers in HT22 cells, and compared whether changes in the expression of serotonergic neuronal phenotype-related proteins in HT 22 cells represented the characteristics of differentiated hippocampal primary neurons. As shown in Figure 2A, primary cultured hippocampal neurons expressed higher levels of SERT, 5-HT_1a_R, and TPH2, which are the rate-limiting enzymes of 5-HT biosynthesis. Similarly, when HT22 cells differentiated, the expression of serotonergic neuronal markers, SERT (F (2, 21) = 7.799, *p* = .0029) 5-HT_1a_R (F (2, 33) = 29.11, *p* < .0001), and TPH2 (F (2, 21) = 13.73, *p* = .0002) were upregulated significantly, similar to that in hippocampal primary cultured neurons (Figures 2B,C).

**FIGURE 3:**
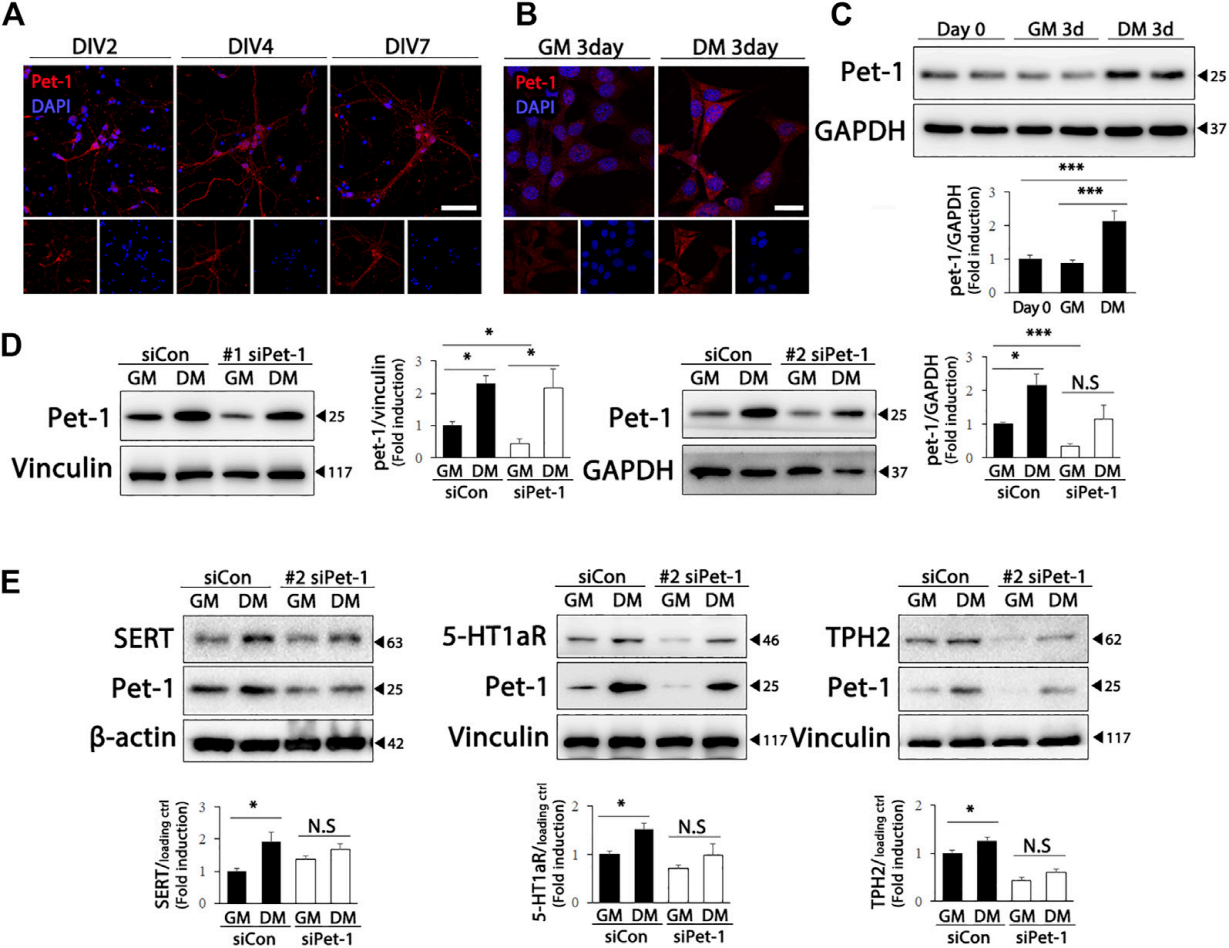
Pet-1 enhances the serotonergic phenotype in differentiated HT22 cells. **(A)** Immunofluorescence staining of Pet-1 (red) in hippocampal primary culture. DAPI staining (blue) indicates the nucleus. Scale bar = 50 μm. **(B,C)** Pet-1 (red) immunofluorescence staining **(B)** and western blotting **(C)** of HT22 cells grown in normal growth medium and differentiation medium for 3 d. DAPI staining (blue) indicates the nucleus. Scale bar = 25 μm. GAPDH is used as loading control. **(D,E)** Control siRNA and Pet-1 siRNA were transiently transfected in HT22 cells, changed with normal growth medium and differentiation medium for 3 d. Western blotting for analyzing Pet-1 **(D)** and SERT, 5-HT_1a_R, and TPH2 **(E)** expression. GAPDH, vinculin, and β-actin were used as loading controls. Data are representative of at least three independent experiments and are quantified using densitometric analysis. Statistical significance: *p < .05, and ***p < .001.

Pet-1, an ETS domain transcription factor, is required for the generation of serotonergic neurons, as well as for the maintenance of serotonergic phenotypes and function in adults (Liu et al., 2010). Therefore, we wondered whether Pet-1 expression increased during differentiation and was involved in the upregulation of serotonergic phenotypes in HT22 cells. As shown in Figure 3A, Pet-1 expression increased after DIV in primary hippocampal cultured neurons. Furthermore, significantly higher Pet-1 (F (2, 39) = 11.82, *p* < .0001) expression was detected in HT22 cells grown in differentiation medium than in cells grown in normal medium (Figures 3B,C). To determine whether Pet-1 was crucial in the differentiation-induced increase in serotonergic features of HT22 cells, we used two Pet-1 siRNAs targeting different regions of the Pet-1 genome (Figure 3D) (siPet-1 #1: F (3, 8) = 10.35, *p* = .0040; siPet-1 #2, F (3, 12) = 7.295, *p* = .0048). The degree of increase in the levels of serotonergic neuronal markers of HT22 cells *via* differentiation was significantly reduced in Pet-1 knockdown HT22 cells compared to those in normal HT22 cells (Figure 3E) (SERT: F (3, 12) = 4.321, *p* = .0277; 5-HT_1a_: F (3, 20) = 6.494, *p* = .0030; TPH2: F (3, 20) = 33.81, *p* < .0001). These findings show that the differentiation of HT22 cells enhances serotonergic neuronal markers *via* Pet-1 regulation.

### 3.3 Differentiated HT22 cells show serotonergic neuronal phenotype

**FIGURE 4:**
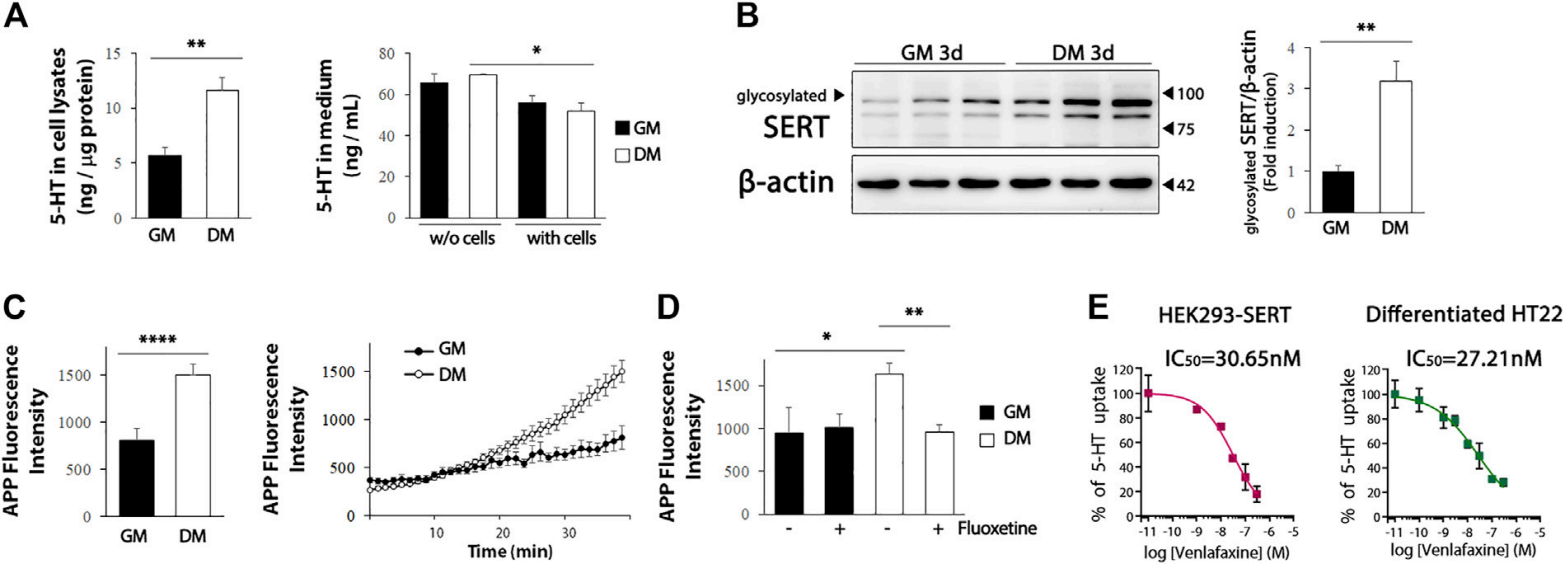
SERT activity in differentiated HT22 cells. **(A)** HT22 cells were grown in normal growth medium and differentiation medium for 3 d, and 5-HT levels were determined in cell lysate (left) and medium (right) using ELISA. **(B)** Western blotting for glycosylated SERT expression in HT22 cells. Beta-actin was used as loading control. **(C)** Accumulation of APP^+^ fluorescence in undifferentiated and differentiated HT22 cells. **(D)** Co-treatment with fluoxetine (10 μM) abolishes accumulation of APP^+^ fluorescence in differentiated HT22 cells. **(E)** Venlafaxine (.1–1 uM) compete for SERT binding [specific for (^3^H) 5-HT binding] in HEK293-SERT and differentiated HT22 cells. The IC_50_ value was generated from each of these curves. Data are representative of at least three independent experiments and are quantified using densitometric analysis. Statistical significance: *p < .05, **p < .01 and ****p < .0001.

We further evaluated whether the increased levels of serotonergic neuronal markers in the differentiated HT22 cells were functional. We detected a significant increase in intracellular 5-HT levels in the differentiated HT22 cells (Figure 4A, left, t = 4.134, df = 9, *p* = .0025), and a decrease in the 5-HT levels in the differentiated medium (Figure 4A, right, t = 2.354, df = 10, *p* = .0404). Five-HT reuptake into cells through SERT is an important regulatory mechanism of serotonergic neurotransmission and is, thus, a major therapeutic target for antidepressants. The efficacy of antidepressants SSRI/SNRI is closely associated with the extent of their inhibition of 5-HT uptake from the extracellular space by targeting SERT (Krout et al., 2017). Therefore, it is important to identify *in vitro* systems capable of measuring SERT activity by using appropriate cells for antidepressant screening. Because glycosylation of SERT favorably regulates transporter conformation and function (Nobukuni et al., 2009; Cooper et al., 2019), we first evaluated whether glycosylated SERT was increased by differentiation. As shown in Figure 4B, glycosylated SERT was significantly increased in the differentiated HT22 cells (t = 4.399, df = 8, *p* = .0023). Next, we analyzed whether the upregulated SERT is functional using APP^+^, which is the fluorescent substrate of SERT (Solis et al., 2012). A significant increase in APP^+^ reuptake was detected in differentiated HT22 cells compared to undifferentiated cells (Figure 4C, t = 4.817, df = 70, *p* < .0001). In addition, fluoxetine, a representative SSRI, attenuated APP^+^ reuptake in differentiated HT22 cells (Figure 4D, t = 3.705, df = 18, *p* = .0016).

We further compared the inhibitory activity of SNRI venlafaxine on SERT between HEK293-SERT and differentiated HT22 cells. HEK293-SERT cells are among the most widely used *in vitro* systems for assessing SERT activity (Manepalli et al., 2011). The SERT inhibition of venlafaxine in these two cell lines was evaluated by measuring [^3^H] 5-HT uptake in the presence of a range of concentrations of venlafaxine. The IC_50_ of venlafaxine in differentiated HT22 cells (IC_50_: 27.21 nM) was comparable to that in HEK293-SERT cells (IC_50_: 30.65 nM). These results indicate that differentiated HT22 cells could be a useful *in vitro* system for 5-HT reuptake assays.

## 4 Discussion

The hippocampus is a complex brain structure implicated in learning, memory, and emotional behavior, and its dysfunction is associated with cognitive decline and several neurological disorders including depression, anxiety, and schizophrenia (Allen et al., 2007; Small et al., 2011; Lieberman et al., 2018). Experimental and clinical evidence suggests that abnormalities in serotoninergic neurotransmission in the hippocampus are associated with increased vulnerability to neuropsychiatric problems. Unfortunately, the lack of *in vitro* models representing functional serotonergic neuronal properties limits our understanding of serotonergic neuron-associated pathologies and associated therapeutic targets. In this study, we demonstrated, for the first time, that differentiated HT22 cells have functional serotonergic properties: increased expression of SERT, TPH2, and 5-HT_1a_R, synthesis of 5-HT, and intracellular uptake of 5-HT *via* SERT.

The Pet-1/E26 transformation-specific transcription factor is a precise marker for serotonergic neurons (Hendricks et al., 2003). Pet-1 plays a crucial role in the transcriptional control of the serotonergic neuron phenotype, which is required for the generation of serotonergic neurons and maintenance of serotonergic phenotypes and function in adults (Hendricks et al., 2003; Liu et al., 2010). Adult Pet-1 null mice have severe deficits in 5-HT_1a_R, TPH2, and SERT in the dorsal raphe nucleus region (Wyler et al., 2016), and show elevated anxiety-like behavior and aggression (Hendricks et al., 2003). The present study demonstrated that differentiated HT22 cells have physiological similarities with serotonin neurons; Pet-1 is upregulated in differentiated HT22 cells, and the increased expression of serotonergic markers *via* differentiation is attenuated in the Pet-1 deficiency condition.

SERT is a 12 transmembrane domain protein that is responsible for 5-HT uptake. 5-HT reuptake *via* SERT is the main process in determining synaptic levels of 5-HT, therefore, SERT is an important target of a class of antidepressants, such as SSRIs and SNRIs. HEK293-SERT cells are commonly used to evaluate the antidepressant efficacy of SSRIs or SNRIs based on the transport kinetics of SERT for 5-HT (Jorgensen et al., 2014). The present study showed that differentiation increased the expression of functional SERT in HT22 cells (Figure 4C). Interestingly, 5-HT reuptake in differentiated HT22 cells was comparable to that in HEK293 cells overexpressing SERT. The IC_50_ of venlafaxine for SERT in differentiated HT22 cells was similar to that in HEK293-SERT cells. Despite the many benefits of *in vitro* studies, such as cost and convenience, these cell models have limitations owing to their inability to replicate an organism’s cellular conditions. The present study proposes a novel *in vitro* model for SSRI development to bridge the gap between *in vitro* and *in vivo* studies. Unlike HEK293-SERT cells, which are non-neuronal cell lines that only overexpress SERT, differentiated HT22 cells are hippocampus-derived and exhibit proteins characteristic of serotonergic neurotransmission, making them an attractive *in vitro* model that reflects the serotonin neurotransmission system *in vivo*. As a result, this cellular model is appropriate for both basic and applied studies on the regulatory mechanisms of serotonin and will improve our understanding of neuropsychiatric disorders caused by serotonin neurotransmission abnormalities.

In summary, the present study showed that differentiated HT22 cells not only show increased expression of cholinergic markers but also the characteristics of serotonergic neurons. As differentiated HT22 cells mimic the environment of real hippocampal neurons more closely, they can be used to overcome the shortcomings of conventional cell line models. Furthermore, this novel *in vitro* model can be used to develop drugs for 5-HT neurotransmission-related diseases.

## Data Availability

The original contributions presented in the study are included in the article/supplementary material, further inquiries can be directed to the corresponding author.
